# Functional improvements desired by patients before and in the first year after total hip arthroplasty

**DOI:** 10.1186/1471-2474-14-243

**Published:** 2013-08-15

**Authors:** Kristi Elisabeth Heiberg, Arne Ekeland, Anne Marit Mengshoel

**Affiliations:** 1 Department of Physiotherapy, Bærum Hospital, Vestre Viken Hospital Trust, Sandvika, Norway; 2 Department of Health Sciences, Institute of Health and Society, University of Oslo, P.O. Box 1089 Blindern, N-0317, Oslo, Norway; 3 Martina Hansen’s Hospital, Sandvika, Norway

**Keywords:** Arthroplasty, Replacement, Hip, Rehabilitation, Desires, Functional improvement, ICF

## Abstract

**Background:**

In the field of rehabilitation, patients are supposed to be experts on their own lives, but the patient’s own desires in this respect are often not reported. Our objectives were to describe the patients’ desires regarding functional improvements before and after total hip arthroplasty (THA).

**Methods:**

Sixty-four patients, 34 women and 30 men, with a mean age of 65 years, were asked to describe in free text which physical functions they desired to improve. They were asked before surgery and at three and 12 months after surgery. Each response signified one desired improvement. The responses were coded according to the International Classification of Functioning, Disability and Health (ICF) to the 1^st^, 2^nd^ and 3^rd^ category levels. The frequency of the codes was calculated as a percentage of the total number of responses of all assessments times and in percentage of each time of assessment.

**Results:**

A total of 333 responses were classified under Part 1 of the ICF, Functioning and Disability, and 88% of the responses fell into the Activities and Participation component. The numbers of responses classified into the Activities and Participation component were decreasing over time (p < 0.001). The categories of Walking (d450), Moving around (d455), and Recreation and leisure (d920) included more than half of the responses at all the assessment times. At three months after surgery, there was a trend that fewer responses were classified into the Recreation and leisure category, while more responses were classified into the category of Dressing (d540).

**Conclusions:**

The number of functional improvements desired by the patients decreased during the first postoperative year, while the content of the desires before and one year after THA were rather consistent over time and mainly concerned with the ability to walk and participate in recreation and leisure activities. At three months, however, there was a tendency that the patients were more concerned about the immediate problems with putting on socks and shoes.

## Background

In the field of rehabilitation, patients are regarded to be experts on their own lives [1]. Many authors maintain that when rehabilitation interventions are being planned, the patients’ own desires regarding functional improvement should be given more weight than is usual today [2]. This means that patients should have a strong say in defining which problems should be addressed during rehabilitation [3], and clinicians should take this into account and tailor the interventions to the patients’ own desires to enable the patients to live meaningful lives [4]. Physiotherapy is a central element in rehabilitation after total hip arthroplasty (THA) for osteoarthritis (OA) [5]. As far as we know, what patients with THA actually want to obtain from physiotherapy is not reported.

Several studies have examined what patients expect from THA surgery. Mancuso et al. [6-8] found that the patients’ preoperative expectations were to obtain pain relief and improve walking [6,7], and these expectations were fulfilled when the patients were asked four years later [8]. The results from other studies not directly examining expectations also suggest that pain relief is obtained and improved physical functioning are reached during the first year after surgery [9-14]. A qualitative study suggests that the patients expect to return to work and their previous level of physical functioning [15]. However, these studies do not especially address what patients expect from rehabilitation or physiotherapy after THA surgery.

Physiotherapy is aimed to improve and optimize physical functioning [16,17]. However, prior studies examining which improvements patients with THA expect with respect to physical functioning is mostly described in rather general terms, for example to improve walking [7]. Some may want to walk safely indoors, while others may want to do more demanding activities, such as skiing or hiking in the mountains, which they enjoyed before they became incapacitated [18]. Thus, we wanted to get a more detailed description of the activities the patients desired to improve during the first year after surgery, and we also wanted to examine whether their desires changed over time.

**Figure 1:**
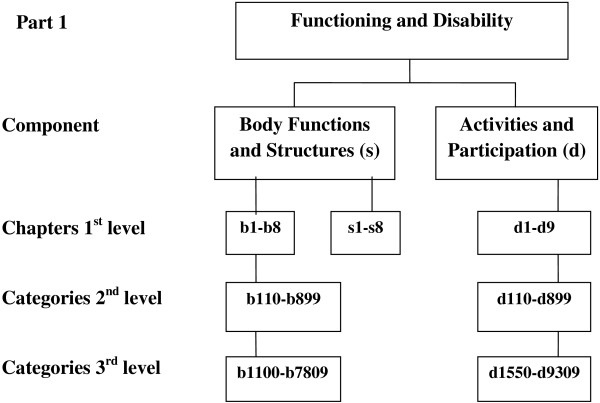
Structure of part 1 of the international classification of functioning, disability and health (icf) applicable to patients after total hip arthroplasty.

A way of assessing patients’ desires is to ask the patients to describe in their own words what they wish to achieve. Such free text responses may be systematised by using the International Classification of Functioning, Disability and Health (ICF), developed by the World Health Organization (WHO). The ICF is a model and classification system that may contribute to broaden our understanding of the different ways in which chronic conditions can affect a patient’s functioning [19]. The ICF model has two parts, each of which contains several components. Part 1 is Functioning and Disability, and includes the components Body Functions and Structures, and Activities and Participation (Figure 1). Part 2, Contextual Factors, also has two components: Environmental Factors and Personal Factors. In the present study we used the ICF as a tool to classify the free-text responses and describe what the patients with THA wished to improve during the first year after surgery.

The objective of this study was to describe the desires of a group of patients regarding improvements in physical functioning before they underwent THA and at three and 12 months after surgery.

## Methods

### Study design and participants

The present study is part of a study designed to examine recovery course the first year after surgery [14] and to examine whether participation in a physiotherapy programme starting three months after surgery influenced the recovery course [20]. The study had a longitudinal design, and the patients were asked to describe what they wanted to improve preoperatively and at three and 12 months postoperatively. Patients with hip OA were consecutively recruited the day before THA surgery and asked to participate in the study. They were recruited from two hospitals in the period from October 2008 to March 2010. The inclusion criteria were a diagnosis of primary hip OA and residence close to the hospital, i.e. within a radius of about 30 km, so as to make it easy for them to attend training sessions. They were excluded if they had OA in a knee or the contralateral hip that restricted walking, a neurological disease, dementia, heart disease, drug abuse and an inadequate ability to read and understand Norwegian. The study was carried out in compliance with the Helsinki Declaration, and formal approval was given by the Regional Committee for Medical Research Ethics and Norwegian Social Science Data Services. Written consent for participation in the study was obtained from those who approved.

### Personal characteristics

Before surgery the patients completed a questionnaire on age, sex, body weight, height, educational level, marital status, comorbidities, history of pain at night, prosthesis in the contralateral hip or knees, and their self-evaluated level of physical activity.

### The patients’ desires regarding improvements in physical functioning

The Patient-Specific Functional Scale (PSFS) has been developed to identify the kinds of problems a particular patient considers to be serious [21-23]. The patient responds in free text to the following question: “Today, are there any activities that you are unable to do or have difficulty with because of your problem?” In the present study we modified the PSFS question as follows “Which activities do you consider it important to improve?” As in the PSFS, the patients were asked to identify one to three activities. The patients were not shown their previous answers in the subsequent assessments at three and 12 months. Whether the question was understandable was tried out among some random patients at the hospital before the study started, and the question seemed understandable for the patients.

### Analysis

All the patients’ desires as expressed in free text were manually coded and classified according to the ICF. The responses were linked to the most closely related ICF categories according to the linking rules [24,25]. Each desire mentioned by each patient was considered to be one response. Thus, a patient who wished to improve three physical functions produced three responses. The desires were first classified under Part 1, Functioning and Disability, or Part 2, Contextual Factors. None of them were found to correspond to Contextual Factors. The desires were then classified under the Body Functions and Structures component or the Activities and Participation component. Then responses were linked first into chapter at 1^st^ level, then category at the 2^nd^ level and the 3^rd^ level [19] (Figure 1). The classification process was completed by the first author in close cooperation with the third author, both being physiotherapists. When they were uncertain or they disagreed, the linking was discussed until consensus was reached. To make the coding process transparent [25], examples of how the responses were linked to the ICF are presented in Table 1. At each assessment, the total number of ICF-coded responses was counted and the proportion of responses in each category was calculated as a percentage of the total number of responses at the particular assessment time. To analyse whether the individuals changed their number of desires over time Friedman Test was used due to non-normally distributed data.

**Table 1:** Examples of patients’ desires of functional improvements linked to the international classification of functioning, disability and health

**2**^**nd **^**level classification**	**3**^**rd **^**level classifiacation**	**Patient’s free text response**
b455: Exercise tolerance functions	b4550: General physical endurance	“Improve endurance”
b730: Muscle power functions	b7301: Power of muscles in one limb	“Improve muscle strength in the limb”
b755: Involuntary movement reaction functions	No code at 3^rd^ level	“Balance”
Walking (d450)	d4500: Walking	“To walk”
	d4501: Walking long distances	“Walking longer distances”
Moving around (d455)	d4551: Climbing	“Walking on stairs”
Dressing (d540)	d5402: Putting on socks and shoes	“Putting on sock and shoes”
		“Socks”
		“Tie shoes”
Caring for household objects (d650)	D6505: Taking care of plants and animals	“Gardening”
Recreation and leisure (d920)	d9201: Sport	“Skiing”
		“Bicycling”
		“Swimming”
		“Playing golf”
		“Playing tennis”
		“Playing badminton”
		“To participate in a training group”
	d9208: Other specified recreation and leisure activities	“Hiking in the mountain”
		“Go for long walks in the woods”
		“Go for walks a couple of hours”
		“Go for long walks with the dog”
		“Hunting”
		“Fishing”
		“Build a cottage”
		“Woodcutting”

## Results

### Participants

Before surgery, 128 patients who fulfilled the inclusion criteria were asked to participate. Thirty-six patients declined, leaving 92 to be assessed preoperatively. Twenty-four patients withdrew from the study at three months, and four withdrew before the 12-month assessments. In this study, we report the responses of the 64 patients who participated at all assessment times. The patients’ mean age was 65 years, range 45–81, and the group included 34 women and 30 men (Table 2).

**Table 2:** Personal characteristics of the patients before total hip arthroplasty (n = 64)

**Characteristics**	**n (%)**	**Mean (95% CI)**
Age (y)		65 (64, 67)
Body mass index		27 (26, 28)
Women	34 (53)	
Educational level of >12 years	37 (58)	
Married/cohabiting	50 (78)	
Exeter prosthesis	47 (73)	
Spectron prosthesis	17 (27)	
Previous prosthesis hip or knee	19 (30)	
Pain at night	50 (78)	
Previous physical activity level (high/moderate)	45 (70)	
Comorbidity	20 (31)	
Physiotherapy within/during first 3 months	46 (71)	

### Overview of the patients’ responses

A total of 333 free-text responses were received at the three assessment times, all of which were classified under the Functioning and Disability part of the ICF. Of these, 41 responses (12%) were classified into six different categories under Body Functions and Structures at the 2^nd^ level (Table 3), while 292 responses (88%) were classified into ten categories under Activities and Participation at the 2^nd^ level (Table 4). The total number of responses at each assessment time decreased during the year, from 145 responses before surgery to 109 at three months and 79 at 12 months.

**Table 3:** No. (% of total) of responses classified into part 1, body functions and structures, of the international classification of functioning, disability and health

**1**^**st **^**level classification (ICF chapters)**	**2**^**nd **^**level classification (ICF categories)**	**3**^**rd **^**level classification (ICF categories)**	**Before surgery no. (% of total 145)**	**3 months after surgery no. (% of total 109)**	**12 months after surgery no. (% of total 79)**
b 1: Mental functions	Sleep functions (b134)	Quality of sleep (b1343)	2 (1.4)	0 (0)	0 (0)
b 4: Functions of cardiovascular and respiratory systems	Exercise tolerance functions (b455)	General physical endurance (b4550)	5 (3.4)	2 (1.8)	1 (1.3)
b 7: Neuromuscular and movement-related functions	Mobility of joint functions (b710)	Mobility of a single joint (b7100)	5 (3.4)	4 (3.7)	1 (1.3)
	Muscle power functions (b730)	Power of muscles in one limb (b7301)	0 (0)	2 (1.8)	2 (2.5)
	Involuntary movement reaction functions (b755)	No code at 3^rd^ level	1 (0.7)	7 (6.4)	8 (10.1)
	Gait pattern function (b770)	No code at 3^rd^ level	1 (0.7)	0 (0)	0 (0)
Total no. of responses of Body Functions and Structures			14 (9.6)	15 (13.7)	12 (15.2)

Differences in number of responses within subjects over time; p = 0.8.

**Table 4:** No. (% of total) of responses classified to part 1, activities and participation, of the international classification of functioning, disability and health

**1**^**st **^**level classification (ICF chapters)**	**2**^**nd **^**level classification (ICF categories)**	**3**^**rd **^**level classification (ICF categories)**	**Before surgery no. (% of total 145)**	**3 months after surgery no. (% of total 109)**	**12 months after surgery no. (% of total 79)**
d 4: Mobility	Changing basic body position (d410)	Lying down (d4100)	3 (2.1)	0 (0)	3 (3.8)
		Squatting (d4101)	0 (0)	3 (2.8)	1 (1.3)
		Sitting (d4103)	4 (2.8)	2 (1.8)	0 (0)
		Bending (d4105)	3 (2.1)	3 (2.8)	2 (2.5)
	Maintaining body position (d415)	Maintaining a kneeling position (d4152)	1 (0.7)	0 (0)	0 (0)
		Maintaining a sitting position (d4153)	1 (0.7)	0 (0)	1 (1.3)
		Maintaining a standing position (d4154)	1 (0.7)	1 (0.9)	0 (0)
	Walking (d450)	Walking (d4500)	22 (15.2)	8 (7.3)	6 (7.6)
		Walking long distances (d4501)	20 (13.8)	22 (20.2)	17 (21.5)
		Walking on different surfaces (d4502)	3 (2.1)	0 (0)	0 (0)
	Moving around (d455)	Crawling (d4550)	0 (0)	1 (0.9)	0 (0)
		Climbing (d4551)	18 (12.4)	17 (15.6)	6 (7.6)
		Running (d4552)	5 (3.4)	1 (0.9)	4 (5.1)
d 5: Self-care	Dressing (d540)	Dressing (d5400)	1 (0.7)	0 (0)	0 (0)
		Putting on socks and shoes (d5402)	9 (6.2)	18 (16.5)	5 (6.3)
d 6: Domestic life	Household tasks (d640)	Cleaning (d6402)	0 (0)	2 (1.8)	1 (1.3)
	Caring for household objects (d650)	Taking care of plants and animals (d6505)	3 (2.1)	2 (1.8)	1 (1.3)
d 8: Major life areas	Work and employment (d845)	Keeping a job (d845)	1 (0.7)	0 (0)	0 (0)
d 9: Community, social and civic life	Recreation and leisure (d920)	Sport (d9201)	10 (6.9)	5 (4.6)	16 (20.3)
		Other specified recreation and leisure activities (d9208)	26 (17.9)	9 (8.3)	4 (5.1)
Total no. of responses of Activities and Participation			131 (90.5)	94 (86.2)	67 (85.0)

Differences in number of responses within subjects over time; p < 0.001.

### Desired improvements of physical functioning

The results are shown in detail in Tables 3 and 4. Of the total responses at the different assessment times, 10% to 15% were classified under the component Body Functions and Structures, while 85% to 91% of the responses were classified into the component Activities and Participation. At the 2^nd^ level classification 42% to 47% of the responses were classified into the categories Walking (d450) and Moving around (d455) at the different time points. Over time, 13% to 25% of the responses were classified into the category Recreation and leisure (d920). At three months there was a tendency of fewer responses coded into the category Recreation and leisure (d920) and some increase of the responses classified into the Dressing (d540) category. At 12 months, 12 patients had no further desires and answered that everything was OK.

When comparing the responses of each individual at the different time points a change in what they wanted to improve from one time to another was seen for most of the patients. The different desires of improvement were distributed evenly across ages and among men and women. The number of desires within patients classified into the Body Functions and Structures component did not change over time (p = 0.8). There was a decrease in number of desires classified into the Activities and Participation component reported by the subjects from preoperative median (25%-75% percentiles) 2 (1–3), to three months 1 (1–2), and to 12 months after surgery 1 (0–1) (p < 0.001).

## Discussion

More than 85% of the patients’ desires before and after THA were classified under the Activities and Participation component of the ICF. More than half of the total responses were classified into the categories of Walking, Moving around, and Recreation and leisure. The desires were rather consistent over time, but there was noticed some reduction of responses in the Recreation and leisure category and an increase into the Dressing category at three months after arthroplasty. The number of desires presented by each individual decreased during the first postoperative year.

Our finding that most of the functional improvement responses fell into the Activities and Participation component is in line with previous research on patients with different forms of non-surgical musculoskeletal disorders. In a large sample of PSFS responses from patients receiving physiotherapy for musculoskeletal disorders, Fairbairn et al. [26] found that most responses could be classified under the activity component of the ICF. Hobbs et al. [27] studied patients’ free text responses to two questions on expectations before THA. One of the questions concerned what the patients felt they needed and the other what they wished to achieve. They found that only a few responses could be classified as Body Functions, and that the majority were classified under the Activities and Participation component. These questions about patients’ needs and desires seem to be closely related to our question about patients’ desires, which suggests that our preoperative results support their findings. In neither of the two studies, however, could any responses be classified at the third category level, so that our study provides a more detailed description of what patients wish to improve before and after surgery. Mancuso et al. [6,8] found that improvements in walking were expected by most of the patients preoperatively. Our results give a more detailed description about the patients’ desire of walking, as the desires of walking and moving about also implied demanding activities such as sport activities and other leisure activities like hunting and fishing. These can be challenging desires to approach for the field of rehabilitation in general and for physiotherapists in particular.

The patients had a decreasing number of desires over time. Further, when looking at each patient’s responses from one assessment to another we found that most of the patients presented new and different desires. This suggests that when improvements were reached in some activities, new desires of improvements within other activities may have appeared. At three months, desires tended to change from recreation and leisure activities to dressing, in particular to put on socks and shoes. This probably reflects the fact that the movement restrictions imposed by the surgeon, which included not allowing hip ROM to exceed 90° of hip flexion during the first three months, made it difficult for them to reach down far enough to put on socks and shoes. At 12 months, these patients no longer seemed to have difficulty with dressing and climbing stairs. However, just like before surgery many of the patients expressed a desire for further improvements classified into the recreation and leisure category. In a previous study of patients with hip and knee OA it was also found that return to recreational activities and no restriction in walking were among the issues of most concern to the patients [28]. The study was based on a questionnaire and only investigated patients’ desires before surgery, while we found that the free text responses related to improvements in recreational and leisure activities were still present at 12 months after surgery. To our knowledge, this is the first study to show that the patients’ desires before surgery remain relatively consistent during the first year after THA.

Questionnaires have been developed to assess therapeutic outcomes from a patient perspective. The Hip Dysfunction and Osteoarthritis Outcome Score (HOOS) [29] and the Harris Hip Score (HHS) [30] are frequently used for assessing outcome after THA. In these questionnaires pain is essential, together with physical functioning. Our question was related to functional improvements desired by the patients and explains why pain relief was not an adequate answer to our question. Both HOOS and HHS mainly address activities related to hip ROM and different forms of indoor everyday activities. We found that many of the issues of physical functioning relevant to the patients are not covered in the questionnaires, such as endurance, balance, and different leisure activities, like hiking in the woods, skiing and bicycling. In the HHS, there are two items out of ten about walking long distances and using public transport, and in the HOOS three items out of 40 that address shopping, running and performing heavy domestic duties. Thus, there is a discrepancy between what our patients wanted to achieve and what is captured by the questionnaires. In the categories under the Activities and Participation component, the questionnaires include many items related to daily activities such as rising up from the bed or a chair, putting on socks and shoes and walking short distances. According to our findings these items can be found relevant by the patients in the short term after surgery, but in less extent 12 months after surgery where the patients seem to focus on more demanding activities. As these particular questionnaires do not deal fully with concerns that patients may find important, it can be difficult to use these instruments when evaluating whether the goals of rehabilitation are reached.

The validity of the results depends on the quality of the process of linking the responses to the ICF. The linking recommendations have been followed [25]. In order to address a question about validity, we have chosen to make our coding process as transparent as possible in Table 1, according to the discussion of Fayed et al. [31]. Several authors have used two independent coders to minimize assessor bias. However, a high reliability between coders has been reported [25,27,32]. In these studies, the reliability was not examined at the 3^rd^ category level. We had few doubts about how to code before we reached to the 3^rd^ level. Especially to the category Recreation and leisure it was often challenging to link the responses at the 3^rd^ level because the codes did not have a high enough level of detail. According to the linking rules responses should not be linked to the code Other specified recreation and leisure activities (d9208). Nevertheless, we did not find any other suitable category to classify responses such as “hiking”, “go for walks in the woods”, “hunting”, and “fishing”. Hence, we chose to use this code. Further, it seemed that the patients had no difficulties in understanding the question raised in the modified PSFS, because they did not ask for explanations, and they gave clear and concise responses to the question.

Another important question to address is whether the patients’ responses are biased by the participation in a training programme aimed to improve walking starting three months after surgery and lasting for about two months. Half of the patients participated in this programme. When we examined the responses of the two groups separately, the percentage of responses coded as Body Functions and Activities and Participation, as well as in the categories of Walking, Moving around, and Recreation and leisure, remained approximately unchanged. Taken together, we think our coding is adequately performed at the component and first two levels, but it can be less valid at the 3^rd^ level.

Another important question is whether our results can be generalised to other THA patient populations. The patients in this study, who had been consecutively recruited to participate in a study investigating the effect of a training programme, had a mean age four years younger than the mean age of THA patients in Norway, they were non-obese, higher educated than the Norwegian population, married, and had a moderate or high level of physical activity before surgery. Thus, our group of patients may have been to some extent selected from among a fairly healthy, physically active population. This may also explain that they wanted to be able to perform rather demanding activities. However, increasing numbers of those undergoing arthroplasty today seem to be relatively healthy, and, as our study points out, many of them wish to lead an active life.

## Conclusions

Linking patients’ responses to the ICF showed a decrease in number of desires over time, and the most frequent functional improvements desired by the patients both before and one year after THA were walking, moving around and participating in rather demanding recreation and leisure activities. In the early postoperative phase, on the other hand, the described pattern of the patients’ desires changed and they were more concerned about improving temporary limitations in physical functioning. The improvements desired by the patients were not covered in the most widely used disease-specific questionnaires.

## Abbreviations

HHS: Harris hip score; HOOS: Hip dysfunction and osteoarthritis outcome score; ICF: International classification of functioning, disability and health; OA: Osteoarthritis; PSFS: Patient-specific functional scale; THA: Total hip arthroplasty.

## Competing interests

The authors declare that they have no competing interests.

## Authors’ contributions

KEH, AE and AMM designed the study. MDH and AGK collected the data. KEH analyzed and drafted the manuscript with regular feedback from AMM. All authors read and approved the final manuscript.

## Pre-publication history

The pre-publication history for this paper can be accessed here:

http://www.biomedcentral.com/1471-2474/14/243/prepub
